# Glucagon-like peptide-1 receptor agonists and pancreatic outcomes in chronic pancreatitis: a real-world cohort study

**DOI:** 10.1186/s12876-026-05125-5

**Published:** 2026-07-16

**Authors:** Arkadeep Dhali, Rick Maity, Fayaz Khan, Jyotirmoy Biswas, Abdul Rafae Faisal

**Affiliations:** 1 School of Medicine, Dentistry and Biomedical Sciences, Queen’s University Belfast, Belfast, UK; 2 Academic Unit of Gastroenterology, Sheffield Teaching Hospitals NHS Foundation Trust, Sheffield, UK; 3 School of Medical Science and Technology, Indian Institute of Technology Kharagpur, Kharagpur, India; 4 Department of Palliative Medicine, Roswell Park Comprehensive Cancer Center, Buffalo, USA; 5 Department of General Medicine, Barasat Government Medical College and Hospital, Barasat, India; 6 Department of General Medicine, CMH Multan Institute of Medical Sciences, Multan, Pakistan

**Keywords:** GLP-1 Receptor Agonists, Chronic Pancreatitis, Pancreatitis, Pancreatic Insufficiency, Cohort

## Abstract

**Goal:**

To evaluate the association between glucagon-like peptide-1 receptor agonists (GLP-1 RA) therapy and pancreatic, gastrointestinal, and mortality outcomes in chronic pancreatitis (CP) using the TriNetX US Collaborative Network.

**Background:**

CP patients face heightened risks of pancreatic insufficiency, cancer, and mortality. There are concerns regarding GLP-1 RA use and adverse pancreatic outcomes.

**Methods:**

In this retrospective cohort study, adults (age ≥ 18 years) with CP (ICD-10-CM K86.0 or K86.1) were stratified into Cohort 1 (CP + GLP-1 RA, *n* = 10,978) and Cohort 2 (CP without GLP-1 RA, *n* = 245,024. After propensity score matching on age, sex, race, and comorbidities, 10,625 patients remained in each cohort. Primary and secondary outcomes were assessed over 3 years via risk analyses and Kaplan-Meier survival analyses.

**Results:**

GLP-1 RA users had significantly lower incidence of exocrine insufficiency (3.1% vs. 6.3%; RR 0.491, 95%CI 0.429–0.562, *p* < 0.001), endocrine insufficiency (6.0% vs. 8.2%; RR 0.742, 95%CI 0.664–0.829, *p* < 0.001), recurrent acute pancreatitis (5.8% vs. 10.5%; RR 0.554, 95%CI 0.487–0.631, *p* < 0.001), pancreatic cancer (2.0% vs. 3.9%; RR 0.498, 95%CI 0.421–0.590, *p* < 0.001), ED visits (17.4% vs. 22.4%; RR 0.778, *p* < 0.001), hospitalizations (18.0% vs. 26.3%; RR 0.684, *p* < 0.001), and gastroparesis (2.3% vs. 3.0%; RR 0.771, *p* = 0.003). Critically, all-cause mortality was 6.9% in GLP-1 RA users versus 16.4% in non-users (RR 0.423, 95%CI 0.390–0.459, *p* < 0.001), with 3-year survival of 88.6% versus 79.1%.

**Conclusions:**

GLP-1 RA use was associated with substantially lower risks of pancreatic and gastrointestinal outcomes, along with improved overall survival, providing observational evidence supporting safety signals.

**Supplementary Information:**

The online version contains supplementary material available at 10.1186/s12876-026-05125-5.

## Introduction

Chronic pancreatitis is a progressive fibro-inflammatory disorder characterized by irreversible structural damage to the pancreas, leading to both exocrine and endocrine dysfunction [1]. Patients experience substantial morbidity from recurrent acute pancreatitis episodes, chronic abdominal pain, and pancreatic insufficiency, with 10-year and 20-year survival rates of approximately 70% and 45%, respectively [2, 3]. Diabetes mellitus develops in 40–70% of patients with chronic pancreatitis, representing type 3c diabetes secondary to pancreatic disease. This form of diabetes is characterized by concurrent insulin and glucagon deficiency, resulting in brittle glycemic control with increased hypoglycemia risk compared to type 1 or type 2 diabetes [4, 5].

Glucagon-like peptide-1 receptor agonists (GLP-1 RAs) have emerged as important therapeutic agents for type 2 diabetes and obesity, demonstrating glucose-lowering efficacy, weight reduction, and cardiovascular benefits [6]. These medications enhance glucose-stimulated insulin secretion, suppress glucagon release, and promote satiety through the activation of GLP-1 receptors on pancreatic beta cells. Beyond their metabolic effects, GLP-1 RAs exhibit anti-inflammatory and pleiotropic properties that extend beyond glycemic control.

Historically, concerns existed regarding the potential association between GLP-1 RA use and pancreatic adverse events, including acute pancreatitis and pancreatic cancer. However, recent large-scale meta-analyses and cardiovascular outcome trials have not substantiated an increased risk of pancreatitis or pancreatic malignancy with GLP-1 RA therapy. Despite the widespread use of GLP-1 RAs and the high prevalence of chronic pancreatitis among patients with metabolic disorders, limited data exist regarding the safety and outcomes of GLP-1 RA therapy in patients with established chronic pancreatitis [7]. Therefore, we conducted this large-scale retrospective cohort study using the TriNetX Research Network to evaluate the association between GLP-1 RA exposure and pancreatic outcomes, healthcare utilization, and mortality among patients with chronic pancreatitis. This study has been reported in line with the STROCSS guidelines [8].

## Methods

### Study design and data source

This retrospective cohort study utilized the TriNetX Research Network, to evaluate the association between GLP-1 RA use and pancreatic outcomes among patients with chronic pancreatitis.

### Study population

Two cohorts were defined using validated diagnostic and medication codes. All patients were at least 18 years of age at the time of cohort entry.

- Cohort 1 (GLP-1 RA Exposure): 10,978 patients with chronic pancreatitis (ICD-10-CM codes K86.0 or K86.1) prescribed at least one GLP-1 RA, including dulaglutide, semaglutide, or tirzepatide, which were the primary GLP-1-based agents captured in the cohort query. Other GLP-1 RAs such as liraglutide and exenatide were not included in this analysis. The index date was defined as the first occurrence when both chronic pancreatitis diagnosis and GLP-1 RA prescription criteria were satisfied.
- Cohort 2 (Comparison): 245,024 patients with chronic pancreatitis without GLP-1 RA exposure (excluding any prescription of GLP-1 RA medications or tirzepatide). The index date was the date of the initial chronic pancreatitis diagnosis.

### Study period and observation window

The observation window spanned from 1 day post-index event to 1,095 days (3 years) post-index event. All outcomes were ascertained within this timeframe. Patients were excluded if their index event occurred more than 20 years before the analysis date.

### Outcome measures

Ten primary outcomes were evaluated using standardized code sets:

(i) Pancreatic Outcomes: Exocrine pancreatic insufficiency (ICD-10-CM K86.81 or K90.3), endocrine pancreatic insufficiency (ICD-10-CM E08 or E13), recurrent acute pancreatitis (ICD-10-CM K85), and pancreatic cancer (ICD-10-CM or ICD-O-3 code C25).
(ii) Healthcare Utilization: Emergency department visits, hospitalizations, and all-cause mortality.
(iii) Other Gastrointestinal Outcomes: Gastroparesis (ICD-10-CM K31.84), composite gastrointestinal malignancies (ICD-10-CM C15-C26), and diabetic ketoacidosis/hyperosmolar hyperglycemic state (ICD-10-CM E10.1, E11.1, or E13.1).

For each outcome, patients with the condition documented prior to the observation window were excluded to ensure assessment of new-onset events.

### Propensity score matching

To minimize confounding by indication and baseline imbalances, 1:1 propensity score matching was performed using demographic and clinical variables, including age, sex, race, and 25 comorbidity indicators (hypertension, chronic kidney disease, diabetes mellitus, obesity, dyslipidemia, tobacco use, atrial fibrillation, chronic obstructive pulmonary disease, and others). Standardized differences after matching were all below 0.075, indicating successful covariate balance. Matching reduced Cohort 2 from 245,024 to 10,625 matched pairs while retaining 10,625 of the original 10,978 Cohort 1 patients.

### Statistical analysis

Two complementary statistical approaches were employed for each outcome:

(i) Incidence Analysis: Risk difference, risk ratio, and odds ratio with 95% confidence intervals were calculated using z-tests. Patients with pre-existing outcomes were excluded from analyses.
(ii) Time-to-Event Analysis: Kaplan-Meier survival analysis with daily time intervals was conducted. The log-rank test was used to evaluate differences in survival distributions. Hazard ratios with 95% confidence intervals were estimated using Cox proportional hazards models. The proportional hazards assumption was tested using platform diagnostics.

All analyses were performed within the TriNetX Analytics platform using validated algorithms. Statistical significance was defined as *p* < 0.05.

## Results

### Patient disposition and follow-up

Before matching, Cohort 1 included 10,978 patients with chronic pancreatitis and GLP-1 RA exposure, while Cohort 2 comprised 245,024 patients with chronic pancreatitis without GLP-1 RA exposure. Following 1:1 propensity score matching, 10,625 patients remained in each cohort.

### Follow-up duration

Before matching, Cohort 1 had a mean follow-up of 598.7 ± 387.8 days with a median of 576 days (interquartile range 817 days). Cohort 2 demonstrated a longer follow-up period of 711.0 ± 438.4 days with a median of 1,010 days (IQR 853 days). After matching, follow-up duration remained similar: Cohort 1 mean 600.3 ± 388.0 days (median 580 days, IQR 820) and Cohort 2 mean 709.9 ± 414.0 days (median 883 days, IQR 801).

### Baseline characteristics

Before Propensity Score Matching, Cohort 1 patients were older (59.5 ± 13.1 vs. 55.7 ± 17.1 years, *p* < 0.001) and more frequently female (54.0% vs. 47.4%, *p* < 0.001). Substantial differences existed in comorbidity burden: type 2 diabetes mellitus (81.7% vs. 21.1%), dyslipidemia (80.2% vs. 28.9%), essential hypertension (82.7% vs. 39.1%), obesity (38.1% vs. 4.5%), and obstructive sleep apnea (36.3% vs. 6.0%) (all *p* < 0.001). Exocrine pancreatic insufficiency was more prevalent in Cohort 1 (6.9% vs. 1.0%, *p* < 0.001). Following matching, baseline characteristics were well-balanced between cohorts. Age distributions converged to 59.6 ± 13.1 and 60.3 ± 14.2 years (*p* < 0.001, standardized difference 0.050). Sex distribution became comparable (53.7% female in Cohort 1 vs. 54.0% in Cohort 2, *p* = 0.741). Comorbidity frequencies were closely aligned: type 2 diabetes mellitus (81.2% vs. 84.4%), hypertension (82.2% vs. 84.1%), dyslipidemia (79.6% vs. 80.6%), and obesity (BMI 30–39: 31.7% in both cohorts). All standardized differences were less than 0.075, confirming adequate balance.

### Exocrine pancreatic insufficiency

**Fig. 1:**
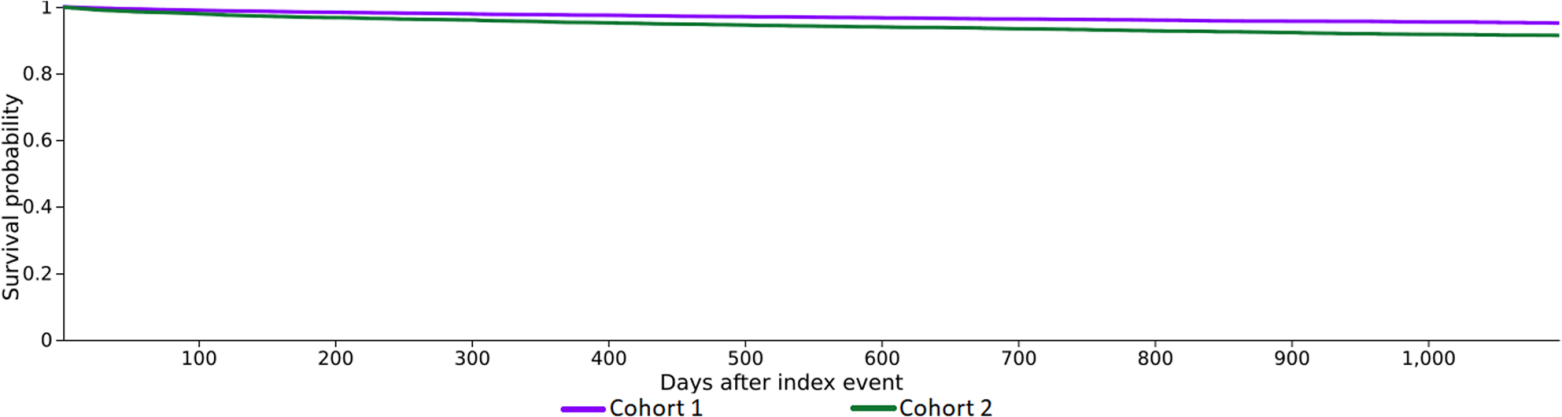
Kaplan-Meier curve showing survival probability without exocrine pancreatic insufficiency, excluding patients with outcome (mortality) prior to the time window

Among patients without prior exocrine insufficiency, 302 of 9,810 Cohort 1 patients (3.1%) developed exocrine pancreatic insufficiency compared to 612 of 9,754 Cohort 2 patients (6.3%). The risk difference was − 3.2% (95% CI -3.8% to -2.6%, z = -10.591, *p* < 0.001). The risk ratio was 0.491 (95% CI 0.429–0.562) and odds ratio 0.474 (95% CI 0.412–0.546). Three-year survival probability without exocrine insufficiency was 95.14% in Cohort 1 versus 91.44% in Cohort 2 (Fig. 1). The hazard ratio was 0.539 (95% CI 0.470–0.619, *p* < 0.001). The log-rank test demonstrated significant differences (χ² = 79.267, *p* < 0.001). The proportional hazards test was not significant (χ² = 1.872, *p* = 0.171), which supports the model assumptions.

### Endocrine pancreatic insufficiency

**Fig. 2:**
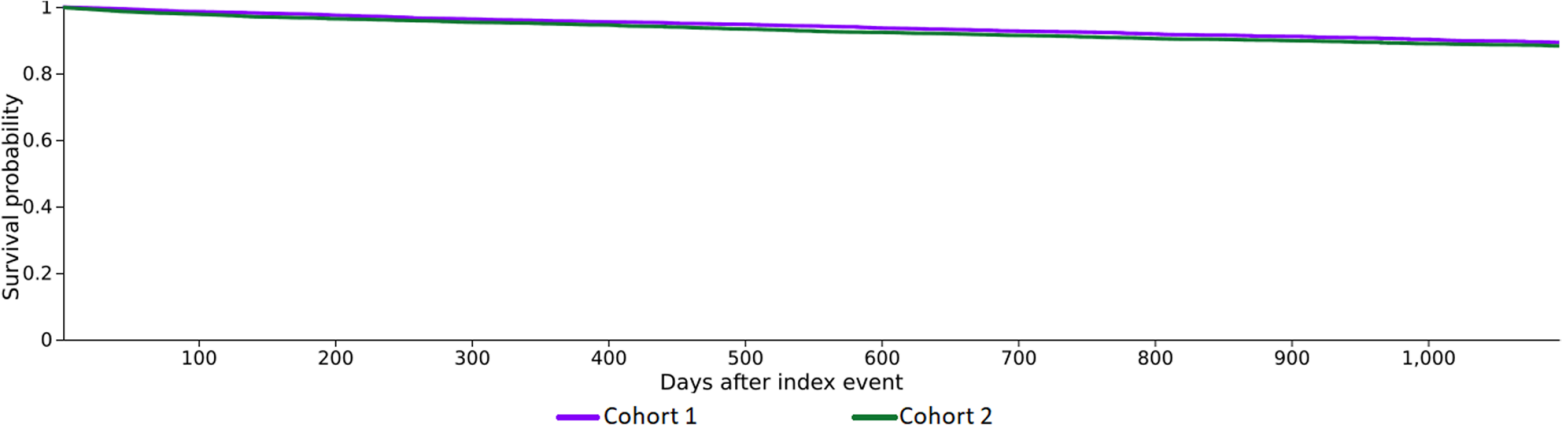
Kaplan-Meier curve showing survival probability without endocrine pancreatic insufficiency, excluding patients with outcome (mortality) prior to the time window

New-onset endocrine insufficiency occurred in 487 of 8,051 Cohort 1 patients (6.0%) and 710 of 8,707 Cohort 2 patients (8.2%). The risk difference was − 2.1% (95% CI -2.9% to -1.3%, z = -5.287, *p* < 0.001). Risk ratio was 0.742 (95% CI 0.664–0.829) and odds ratio 0.725 (95% CI 0.643–0.817). Three-year survival probability was 89.33% in Cohort 1 versus 88.30% in Cohort 2 (Fig. 2). The hazard ratio was 0.851 (95% CI 0.758–0.956, *p* = 0.005). The log-rank test was significant (χ² = 7.435, *p* = 0.006).

### Recurrent acute pancreatitis

**Fig. 3:**
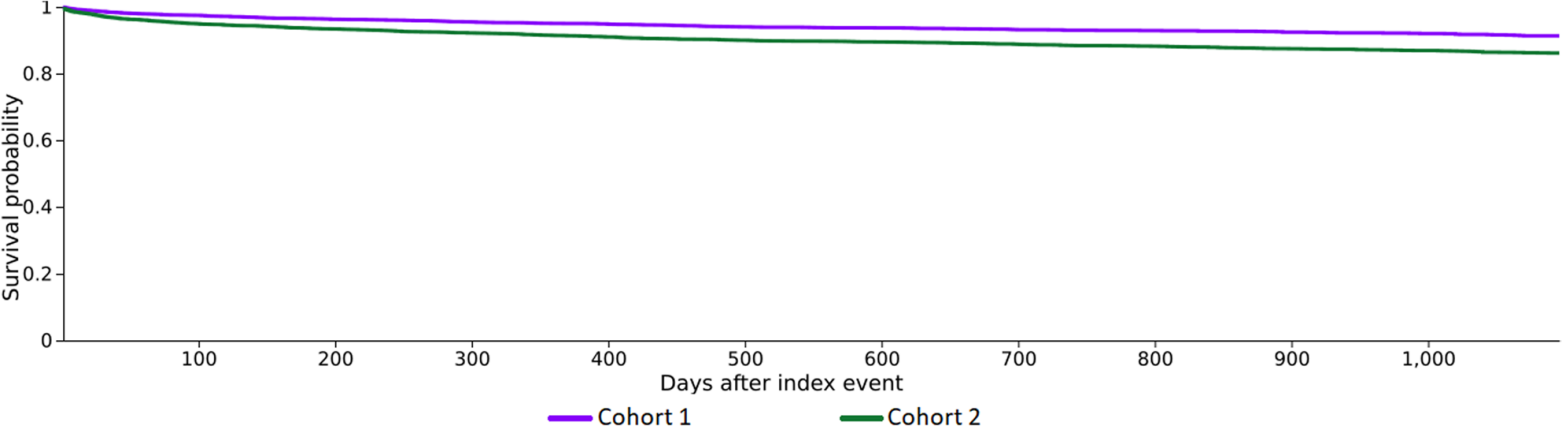
Kaplan-Meier curve showing survival probability without recurrent acute pancreatitis, excluding patients with outcome (mortality) prior to the time window

Among patients without prior episodes of acute pancreatitis, 329 of 5,639 Cohort 1 patients (5.8%) experienced recurrent acute pancreatitis, compared to 579 of 5,499 Cohort 2 patients (10.5%). The risk difference was − 4.7% (95% CI -5.7% to -3.7%, z = -9.053, *p* < 0.001). Risk ratio was 0.554 (95% CI 0.487–0.631) and odds ratio 0.526 (95% CI 0.457–0.606). Three-year survival probability without recurrent acute pancreatitis was 91.34% in Cohort 1 versus 86.16% in Cohort 2 (Fig. 3). The hazard ratio was 0.584 (95% CI 0.510–0.669, *p* < 0.001). The log-rank test demonstrated significant separation (χ² = 61.836, *p* < 0.001).

### Pancreatic cancer

**Fig. 4:**
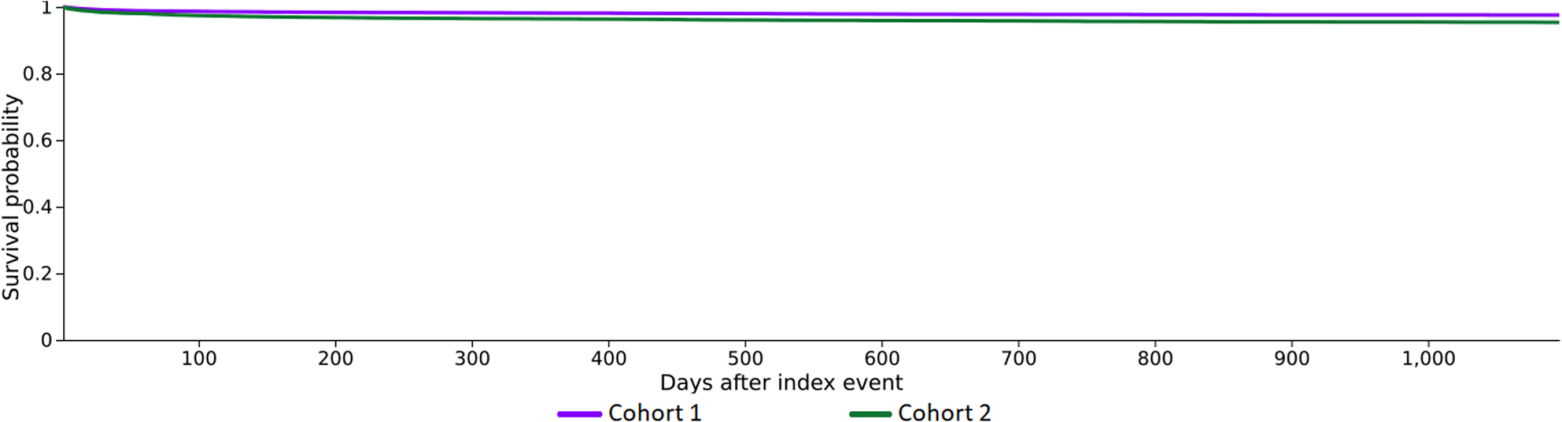
Kaplan-Meier curve showing survival probability without pancreatic cancer, excluding patients with outcome (mortality) prior to the time window

Pancreatic cancer developed in 199 of 10,165 Cohort 1 patients (2.0%) and 388 of 9,878 Cohort 2 patients (3.9%). The risk difference was − 2.0% (95% CI -2.4% to -1.5%, z = -8.271, *p* < 0.001). Risk ratio was 0.498 (95% CI 0.421–0.590) and odds ratio 0.488 (95% CI 0.411–0.581). Three-year survival probability without pancreatic cancer was 97.56% in Cohort 1 versus 95.36% in Cohort 2 (Fig. 4). The hazard ratio was 0.518 (95% CI 0.436–0.614, *p* < 0.001). The log-rank test was highly significant (χ² = 58.965, *p* < 0.001).

### Emergency department visits

Among patients without prior ED visits, 699 of 4,014 Cohort 1 patients (17.4%) had ED encounters compared to 974 of 4,354 Cohort 2 patients (22.4%). Risk difference was − 5.0% (95% CI -6.7% to -3.3%, z = -5.663, *p* < 0.001). Risk ratio was 0.778 (95% CI 0.713–0.849) and odds ratio 0.732 (95% CI 0.657–0.815). Three-year survival probability without ED visits was 72.48% in Cohort 1 versus 70.25% in Cohort 2. Hazard ratio was 0.850 (95% CI 0.771–0.937, *p* < 0.001). Log-rank test was significant (χ² = 10.683, *p* = 0.001).

### All-cause mortality

Death occurred in 735 of 10,581 Cohort 1 patients (6.9%) and 1,737 of 10,583 Cohort 2 patients (16.4%). Risk difference was − 9.5% (95% CI -10.3% to -8.6%, z = -21.439, *p* < 0.001). Risk ratio was 0.423 (95% CI 0.390–0.459) and odds ratio 0.380 (95% CI 0.347–0.416). Three-year survival probability was 88.58% in Cohort 1 versus 79.08% in Cohort 2. Hazard ratio was 0.472 (95% CI 0.433–0.515, *p* < 0.001). Log-rank test showed highly significant separation (χ² = 302.634, *p* < 0.001).

### Hospitalizations

Among patients without prior hospitalizations, 617 of 3,432 Cohort 1 patients (18.0%) were hospitalized compared to 799 of 3,041 Cohort 2 patients (26.3%). Risk difference was − 8.3% (95% CI -10.3% to -6.3%, z = -8.058, *p* < 0.001). Risk ratio was 0.684 (95% CI 0.623–0.751) and odds ratio 0.615 (95% CI 0.546–0.693). Three-year survival without hospitalization was 71.64% in Cohort 1 versus 67.43% in Cohort 2. Hazard ratio was 0.756 (95% CI 0.680–0.840, *p* < 0.001). Log-rank test was significant (χ² = 27.198, *p* < 0.001).

### Gastroparesis

New-onset gastroparesis developed in 227 of 9,831 Cohort 1 patients (2.3%) and 294 of 9,819 Cohort 2 patients (3.0%). Risk difference was − 0.7% (95% CI -1.1% to -0.2%, z = -2.989, *p* = 0.003). Risk ratio was 0.771 (95% CI 0.650–0.915) and odds ratio 0.766 (95% CI 0.643–0.913). The three-year survival probability without gastroparesis was 96.46% in Cohort 1, compared to 95.82% in Cohort 2. Hazard ratio was 0.856 (95% CI 0.719–1.018, *p* = 0.078). The log-rank test approached significance (χ² = 3.102, *p* = 0.078).

### Composite gastrointestinal cancers

Any GI cancer developed in 275 of 9,820 Cohort 1 patients (2.8%) and 466 of 9,479 Cohort 2 patients (4.9%). Risk difference was − 2.1% (95% CI -2.7% to -1.6%, z = -7.647, *p* < 0.001). Risk ratio was 0.570 (95% CI 0.492–0.659) and odds ratio 0.557 (95% CI 0.479–0.649). Three-year survival probability without GI cancer was 96.23% in Cohort 1 versus 93.96% in Cohort 2. Hazard ratio was 0.605 (95% CI 0.521–0.703, *p* < 0.001). Log-rank test was significant (χ² = 44.393, *p* < 0.001).

### Diabetic ketoacidosis/hyperosmolar hyperglycemic state

DKA/HHS occurred in 181 of 9,784 patients in Cohort 1 (1.8%) and in 237 of 9,793 patients in Cohort 2 (2.4%). Risk difference was − 0.6% (95% CI -1.0% to -0.2%, z = -2.759, *p* = 0.006). Risk ratio was 0.764 (95% CI 0.631–0.926) and odds ratio 0.760 (95% CI 0.625–0.924). Three-year survival probability without DKA/HHS was 96.69% in Cohort 1 versus 96.46% in Cohort 2. Hazard ratio was 0.888 (95% CI 0.731–1.079, *p* = 0.231). The log-rank test was not significant (χ² = 1.432, *p* = 0.231).

**Fig. 5:**
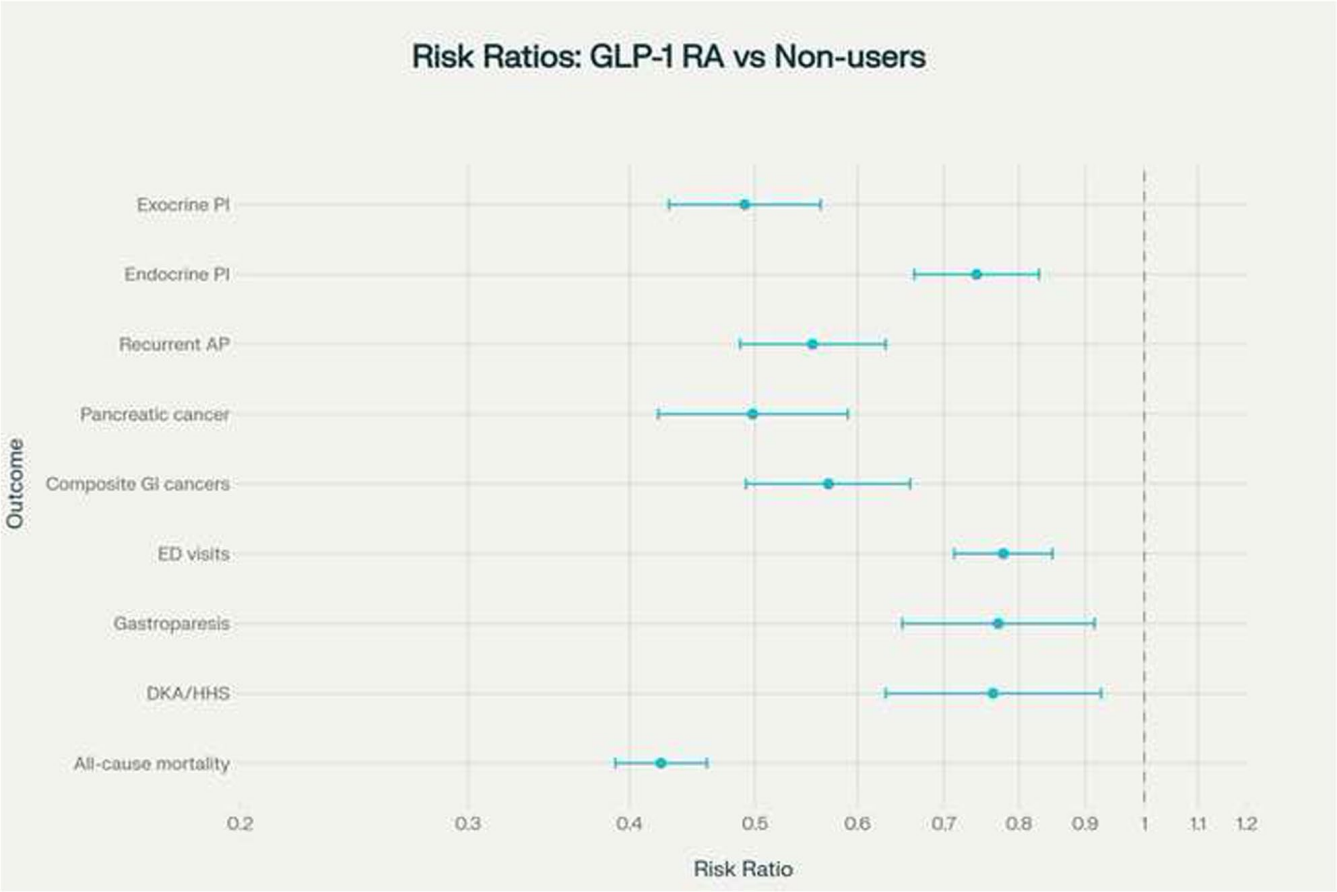
Risk ratios (with 95% confidence intervals) of key pancreatic outcomes and all-cause mortality in GLP-1 RA users versus non-users with chronic pancreatitis

Key pancreatic outcomes and all-cause mortality in GLP-1 RA users versus non-users with chronic pancreatitis have been elucidated in Table 1; Fig. 5.

**Table 1:** Key pancreatic outcomes and all-cause mortality in GLP-1 RA users versus non-users with chronic pancreatitis

Outcome	Cohort	Number at Risk	Number of Events (%)	Risk Difference (95% CI)	Risk Ratio (95% CI)	Odds Ratio (95% CI)	*P*-value
Exocrine pancreatic insufficiency	GLP-1 RA	9,810	302 (3.1%)	−0.032 (− 0.038, −0.026)	0.491 (0.429–0.562)	0.474 (0.412–0.546)	< 0.001
	No GLP- 1 RA	9,754	612 (6.3%)				
Endocrine pancreatic insufficiency	GLP-1 RA	8,051	487 (6.0%)	−0.021 (− 0.029, −0.013)	0.742 (0.664–0.829)	0.725 (0.643–0.817)	< 0.001
	No GLP- 1 RA	8,707	710 (8.2%)				
Recurrent acute pancreatitis	GLP-1 RA	5,639	329 (5.8%)	−0.047 (− 0.057, −0.037)	0.554 (0.487–0.631)	0.526 (0.457–0.606)	< 0.001
	No GLP- 1 RA	5,499	579 (10.5%)				
Pancreatic cancer	GLP-1 RA	10,165	199 (2.0%)	−0.020 (− 0.024, −0.015)	0.498 (0.421–0.590)	0.488 (0.411–0.581)	< 0.001
	No GLP- 1 RA	9,878	388 (3.9%)				
Emergency department visits	GLP-1 RA	4,014	699 (17.4%)	−0.050 (− 0.067, −0.033)	0.778 (0.713_0.849)	0.732 (0.657–0.815)	< 0.001
	No GLP- 1 RA	4,354	974 (22.4%)				
Gastroparesis	GLP-1 RA	9,831	227 (2.3%)	−0.007 (− 0.011, −0.002)	0.771 (0.650–0.915)	0.766 (0.643–0.913)	0.003
	No GLP- 1 RA	9,819	294 (3.0%)				
Diabetic ketoacidosis/ HHS	GLP-1 RA	9,784	181 (1.8%)	−0.006 (− 0.010, −0.002)	0.764 (0.631–0.926)	0.760 (0.625–0.924)	0.006
	No GLP- 1 RA	9,793	237 (2.4%)				
All-cause mortality	GLP-1 RA	10,581	735 (6.9%)	−0.095 (− 0.103, −0.086)	0.423 (0.390–0.459)	0.380 (0.347–0.416)	< 0.001
	No GLP- 1 RA	10,583	1,737 (16.4%)				

## Discussion

This large retrospective cohort study of 21,250 propensity-score matched patients with chronic pancreatitis demonstrates that GLP-1 RA exposure was associated with substantially reduced risks of multiple adverse pancreatic outcomes, decreased healthcare utilization, and markedly lower all-cause mortality over a three-year follow-up period. Specifically, GLP-1 RA use was associated with approximately 50% risk reduction for exocrine pancreatic insufficiency (HR 0.539, 95% CI 0.470–0.619), recurrent acute pancreatitis (HR 0.584, 95% CI 0.510–0.669), and pancreatic cancer (HR 0.518, 95% CI 0.436–0.614), alongside a 15% relative risk reduction for endocrine pancreatic insufficiency (HR 0.851, 95% CI 0.758–0.956). All-cause mortality was reduced by more than half among GLP-1 RA users (HR 0.472, 95% CI 0.433–0.515). However, these findings should be interpreted cautiously, as observational studies cannot establish causality or protective effects.

### Pancreatic outcomes

Our finding of reduced exocrine pancreatic insufficiency with GLP-1 RA therapy aligns with emerging mechanistic data. A randomized controlled trial demonstrated that both short-acting and long-acting GLP-1 RAs significantly improved markers of exocrine pancreatic function in patients with type 2 diabetes, with increased fecal elastase levels and improved fat-soluble vitamin absorption [9]. The mechanisms underlying this beneficial effect may involve GLP-1-mediated enhancement of pancreatic regeneration, reduction in pancreatic inflammation, and preservation of acinar cell function [10, 11].

The substantial reduction in recurrent acute pancreatitis observed in our study contradicts early safety concerns. Meta-analyses have found that GLP-1 RA use was associated with lower lifetime risk of pancreatitis compared to non-users, and both semaglutide and tirzepatide demonstrated favorable outcomes regarding acute pancreatitis recurrence [12, 13]. These findings suggest that GLP-1 RAs do not exacerbate pancreatic inflammation and may possess protective properties through anti-inflammatory mechanisms.

The anti-inflammatory effects of GLP-1 RAs are well-documented and particularly relevant in chronic pancreatitis, where ongoing inflammation drives disease progression [14]. GLP-1 RAs suppress production of pro-inflammatory cytokines including TNF-α, IL-6, and IL-1β, reduce NF-κB activation, and promote anti-inflammatory pathways including AMPK [15, 16]. These properties may attenuate disease progression and reduce acute exacerbations.

Our observation of significantly reduced pancreatic cancer risk among GLP-1 RA users (HR 0.518) is supported by recent studies. Multiple large cohort studies have found no association between GLP-1 RA treatment and pancreatic cancer incidence, while some have reported a 53% reduced risk compared to insulin therapy [17, 18]. These findings likely reflect the metabolic benefits of GLP-1 RAs, including improved glycemic control and weight reduction, both of which are established risk factors for pancreatic malignancy. Additionally, interventions that attenuate pancreatic inflammation may reduce cancer progression in chronic pancreatitis, which confers a 4% cumulative risk of pancreatic cancer over 15–20 years [19]. That being said, the observed association between GLP-1 RA use and lower pancreatic cancer incidence should be interpreted cautiously. Given the observational design, this finding may reflect residual confounding, surveillance bias, or differences in diagnostic intensity rather than a true protective effect. Because pancreatic cancer typically develops over a long latency period, the 3-year observation window may be insufficient to establish meaningful causal inference. Therefore, this finding should be considered hypothesis-generating and requires validation in prospective long-term studies.

### Endocrine function and metabolic outcomes

The modest but significant reduction in endocrine pancreatic insufficiency (HR 0.851) likely reflects GLP-1 RA effects on beta cell function. GLP-1 RAs enhance beta cell survival by promoting proliferation, inhibiting apoptosis, and reducing oxidative stress [10, 20]. Recent evidence demonstrates that pancreatic alpha cells can produce bioactive GLP-1, particularly under diabetic conditions, suggesting autocrine and paracrine signalling mechanisms that support beta cell function [21, 22]. In patients with chronic pancreatitis and compromised islet mass, preservation of remaining beta cells through GLP-1 RA therapy may delay diabetes development. The glucose-dependent mechanism of action also confers a lower risk of hypoglycemia compared to insulin, an important consideration given the brittle diabetes phenotype of type 3c diabetes [5, 23].

### Healthcare utilization and mortality

The substantial reductions in hospitalizations (HR 0.756), emergency department visits (HR 0.850), and all-cause mortality (HR 0.472) likely reflect multiple mechanisms. The cardiovascular benefits of GLP-1 RAs are well established, with meta-analyses demonstrating a 13% reduction in major adverse cardiovascular events and a 13% reduction in all-cause mortality in patients with type 2 diabetes [6, 24]. Given that cardiovascular disease represents a leading cause of death in patients with chronic pancreatitis, the cardioprotective effects—mediated through blood pressure reduction, lipid profile improvement, anti-inflammatory effects, and direct myocardial protection—likely contribute substantially to the observed mortality benefit [2, 25].

Additionally, a reduction in pancreatic-related complications and hospitalizations for acute pancreatitis exacerbations directly decreases healthcare utilization. The weight loss achieved with GLP-1 RA therapy (15–25% body weight reduction) contributes to reduced metabolic stress, improved insulin sensitivity, and decreased visceral adiposity-related inflammation [26, 27]. The magnitude of the observed mortality benefit (a 56.2% relative risk reduction) is noteworthy and exceeds that reported in cardiovascular outcome trials of GLP-1 RAs in general diabetic populations. It may be attributed, at least in part, to confounding by indication and the healthy-user effect, as the GLP-1 RA cohort may have better access to health care or closer monitoring. However, the observed mortality reduction cautiously suggests that the benefits may be particularly pronounced in patients with chronic pancreatitis. Lending itself to cautious interpretation, this may reflect the high baseline mortality risk in chronic pancreatitis and multiple mechanisms through which GLP-1 RAs confer benefit.

### Gastroparesis and other gastrointestinal outcomes

The modest reduction in gastroparesis (HR 0.856, *p* = 0.078) warrants discussion, given the known effects of GLP-1 RA on gastric emptying. While GLP-1 RAs delay gastric emptying, in patients with diabetes, gastroparesis is often related to long-standing hyperglycemia and autonomic neuropathy rather than medication effects [28]. Symptoms associated with GLP-1 RAs are typically transient and reversible upon dose adjustment. Our finding of non-increased gastroparesis risk suggests that the benefits of improved glycemic control may offset direct effects on gastric motility.

The significant reduction in composite gastrointestinal malignancies (HR 0.605) extends beyond pancreatic cancer and likely reflects broader anti-inflammatory and metabolic benefits. Chronic inflammation is a well-established driver of carcinogenesis throughout the gastrointestinal tract, and the anti-inflammatory properties of GLP-1 RA may confer protective effects [29]. Additionally, weight loss and metabolic improvements reduce the risk of obesity-related cancer [22, 30].

### Diabetic ketoacidosis risk

The small but significant reduction in diabetic ketoacidosis/hyperosmolar hyperglycemic state (HR 0.760, *p* = 0.006) is reassuring given concerns about GLP-1 RA-associated ketoacidosis, particularly when insulin is rapidly reduced [31, 32]. Most reported cases occur when concomitant insulin is abruptly discontinued rather than tapered. In patients with type 3c diabetes, insulin dependence is common, and GLP-1 RAs should be viewed as adjunctive therapy [4]. Our findings suggest that when used appropriately in patients with chronic pancreatitis, GLP-1 RAs do not increase the risk of ketoacidosis and may improve metabolic stability through enhanced glycemic control.

### Mechanisms of benefit

The beneficial effects of GLP-1 RAs in chronic pancreatitis likely involve multiple pathways. First, anti-inflammatory effects mediated through suppression of pro-inflammatory cytokines and NF-κB signaling may attenuate the ongoing inflammatory process that drives fibrosis and disease progression [10, 33]. Second, GLP-1 may enhance pancreatic regeneration through stimulation of beta cell proliferation and differentiation, preserving remaining pancreatic function [10, 33]. Third, improved glycemic control and weight reduction decrease systemic inflammation and metabolic stress exacerbating pancreatic damage [6, 28]. GLP-1 RAs, through their effects on glucose and lipid metabolism and their anti-inflammatory properties, may modulate pancreatic stellate cell activation and reduce fibrogenesis [11, 16]. However, due to the study’s observational design, these mechanisms cannot be fully elucidated. Further basic research and prospective clinical studies are needed to clarify how GLP-1 RAs contribute to improved outcomes in CP. A better understanding of these mechanisms may substantially change the management of CP.

### Study limitations

First, as an observational study, causality cannot be established, and unmeasured confounding cannot be entirely excluded despite robust propensity score matching [34, 35]. Factors such as disease severity, alcohol consumption patterns, and medication adherence could influence outcomes. Second, TriNetX database’s reliance on ICD-10 diagnostic coding may be subject to misclassification, particularly for outcomes such as pancreatic cancer and pancreatic insufficiency, where sensitivity may be limited. Third, we lacked granular data on GLP-1 RA dosing, duration, treatment persistence, adherence, and specific agents. Patients who are intolerant of therapy may discontinue early, while others may remain on long-term treatment. This exposure heterogeneity may introduce misclassification bias and affect the observed associations. Fourth, exclusion of certain GLP-1 RAs such as liraglutide and exenatide may limit generalizability across the entire drug class. Fifth, differential index date definitions between exposed and unexposed cohorts and shorter mean follow-up in the GLP-1 RA cohort (600 vs. 710 days) may introduce immortal time bias and artificially lower all event rates, though time-to-event analyses account for differential follow-up. Finally, confounding by indication remains a consideration, as GLP-1 RA users likely represent a selected population. Our study included both alcohol-induced and other chronic pancreatitis, but could not stratify outcomes by etiology. The three-year observation window may not fully capture long-term disease trajectories [36, 37].

## Conclusion

Our findings provide reassurance regarding the safety of GLP-1 RA therapy in chronic pancreatitis, challenging historical concerns about pancreatic adverse events. They suggest that GLP-1 RAs may confer substantial benefits beyond glycemic control, including reduction in disease complications and mortality. Given the high prevalence of diabetes in chronic pancreatitis (40–70%), GLP-1 RAs represent a valuable therapeutic option addressing multiple aspects of disease pathophysiology. The observed benefits must be balanced against known adverse effects, including gastrointestinal symptoms that may be troublesome in patients with chronic pancreatitis. Given the observational design and potential biases, these findings should not be interpreted as causal or practice-changing. Nevertheless, in appropriately selected patients with chronic pancreatitis and coexisting diabetes or obesity, GLP-1 RAs offer a favorable risk-benefit profile. Future prospective randomized controlled trials are needed to definitively establish efficacy and safety, identify patient subgroups most likely to benefit, and elucidate mechanisms underlying the observed benefits.

## Supplementary Information

Supplementary Material 1.

## Data Availability

This study analysed data from TriNetX, a global federated health research network providing access to de-identified electronic medical records from participating healthcare organizations. Thus, the generated dataset is not openly available to researchers outside the study team and can be obtained from the corresponding author upon reasonable request.

## Abbreviations

CI: Confidence interval
CP: Chronic pancreatitis
DKA: Diabetic ketoacidosis
ED: Emergency department
GI: Gastrointestinal
GIP: Glucose-dependent insulinotropic polypeptide
GLP-1: Glucagon-like peptide-1
GLP-1 RA: Glucagon-like peptide-1 receptor agonist
HHS: Hyperosmolar hyperglycemic state
HR: Hazard ratio
ICD-10-CM: International Classification of Diseases, Tenth Revision, Clinical Modification
ICD-O-3: International Classification of Diseases for Oncology, Third Edition
IQR: Interquartile range
IRB: Institutional review board
NF-κB: Nuclear factor kappa B
PSM: Propensity score matching
RA: Receptor agonist
RCT: Randomized controlled trial
RR: Risk ratio
STROCSS: Strengthening the Reporting of Cohort, Cross-Sectional and Case-Control Studies in Surgery
TNF-α: Tumor necrosis factor alpha
